# Long-Term Genome Monitoring Retraces the Evolution of Novel Emerging Porcine Reproductive and Respiratory Syndrome Viruses

**DOI:** 10.3389/fmicb.2022.885015

**Published:** 2022-04-13

**Authors:** Lirun Xiang, Hu Xu, Chao Li, Yan-Dong Tang, Tong-Qing An, Zhen Li, Chunxiao Liu, Shuaijie Song, Jing Zhao, Chaoliang Leng, Xiangyang Qu, Yingjun Sun, Jinmei Peng, Qian Wang, Xuehui Cai, Zhi-Jun Tian, Hongliang Zhang

**Affiliations:** ^1^State Key Laboratory of Veterinary Biotechnology, Harbin Veterinary Research Institute, Chinese Academy of Agricultural Sciences, Harbin, China; ^2^Henan Key Laboratory of Insect Biology in Funiu Mountain, Henan Provincial Engineering Laboratory of Insects Bio-Reactor, China-UK-NYNU-RRes Joint Laboratory of Insect Biology, Nanyang Normal University, Nanyang, China; ^3^Hanswine FoodGroupCo., Ltd., Maanshan, China

**Keywords:** PRRSV, novel strain, re-recombination, evolution, genetic diversity

## Abstract

Porcine reproductive and respiratory syndrome virus (PRRSV) causes tremendous economic losses to the swine industry worldwide. In China, novel PRRSVs have frequently emerged in recent years, but the evolutionary relationship among these viruses has remained unclear. In the present study, a 4-year PRRSV genome-monitoring study was performed on samples from a pig farm. We observed that NADC30-like PRRSVs with higher mutation rates replaced HP-PRRSVs as the epidemic strains. We monitored the variation in the same PRRSV strain evolved in a pig herd over 2 years and observed that the low genomic similarity of NADC30-like PRRSVs results from rapid mutation. We also showed that recombination events between NADC30-like and QYYZ-like PRRSVs resulted in the complex recombination patterns of PRRSVs, which have formed gradually over time. Furthermore, recombination of the same strain can occur at different locations and increase the diversity of recombination events. Overall, these findings interpret the evolutionary patterns of novel and emerging PRRSVs, information that is crucial for PRRSV control.

## Introduction

Porcine reproductive and respiratory syndrome (PRRS), one of the most important diseases with economic significance and welfare importance for the swine industry globally, is caused by PRRS virus (PRRSV), an enveloped, positive-sense, single-stranded RNA virus belonging to the genus Betaarterivirus, family Arteriviridae, and order Nidovirales (Kuhn et al., 2016; Brinton et al., 2021). Regarding their genetic diversity, PRRSV is currently classified into two distinct species, Betaarterivirus suid 1 (PRRSV-1) and Betaarterivirus suid 2 (PRRSV-2; Wensvoort et al., 1991; Collins et al., 1992; Brinton et al., 2021). The earliest evidence of PRRSV infection in domestic pigs can be traced back to the mid-1980s in Europe (Grebennikova et al., 2004) and to 1979 in North America (Carman et al., 1995). Although several hypotheses for the origins of PRRSVs have been proposed, the source of these viruses remains unclear due to a lack of conclusive evidence. Interestingly, novel PRRSVs have continually emerged in recent years, which raises the following questions: where are these novel PRRSVs coming from, and how do they evolve?

Based on the ORF5 gene, type 2 PRRSVs are divided into nine lineages (Shi et al., 2010; Gao et al., 2017; Zhang et al., 2018). In mainland China, the PRRSV strain CH-1a, which belongs to sublineage 8.7, was first isolated in 1996. Since 2006, HP-PRRSV (lineage 8.7; Tian et al., 2007; Tong et al., 2007), QYYZ-like PRRSV (lineage 3; Lu et al., 2015), NADC30-like PRRSV (lineage 1; Zhao et al., 2015; Zhou et al., 2015; Sun et al., 2016), NADC34-like PRRSV (lineage 1; Zhang et al., 2018; Xie et al., 2020), and a vaccine revertant PRRSV (JXA1-R-like, lineage 8.7; Jiang et al., 2015) have emerged in mainland China. In recent years, several novel PRRSVs been identified in China that formed through recombination of three or four of the above-mentioned PRRSV lineages (Zhou et al., 2018a; Liu et al., 2019). Unfortunately, the evolutionary relationships among these strains are complex due to their different recombination patterns and low sequence similarity.

Therefore, in the present study, we interpreted the emergence and evolution of PRRSVs on farms of the same large-scale pig producer in China by continuous monitoring for several years and observed that the importation, variation, and recombination of PRRSVs are important causes of the emergence of novel PRRSV strains. In addition, NADC30-like PRRSVs with higher nucleotide substitution and recombination rates are the current epidemic strains. Notably, we observed that NADC30-like and QYYZ-like PRRSVs are the root resulting in recombination and demonstrate that the currently observed complex recombination patterns of PRRSVs formed gradually over time.

## Materials and Methods

### Pig Producer Information and Study Design

Pigs are introduced to the parental pig breeding farms once or twice a year from cooperative pig farms after rigorous screening for PRRSV antigens and antibodies. Pigs at these pig breeding farms are vaccinated over 10 years using RespPRRS MLV (L5), which was developed by serial passage of ATCC VR2332 on MA104 cells (Yuan et al., 2001). In August 2014, we tested for PRRSV from serum and lung tissue samples from all of the pig farms of the investigated pig producer and subsequently tested for PRRSV from serum every 3–4 months. We also detected PRRSV, CSFV, and PRV from serum or lung tissue samples once there were cases of illness or death of suspected infection with PRRSV.

### RT-PCR and Sequence Analysis

Clinical sample disposal and RT-PCR were conducted as described previously (Zhang et al., 2019). The ORF5 and partial NSP2 gene of the positive samples were sequenced, and representative samples were selected for complete genome sequencing. Detailed information regarding the PCR primers used in the present study was described in previous studies (Leng et al., 2014; Zhang et al., 2018, 2019). The gene and complete genome sequencing was conducted as described previously (Zhang et al., 2019). Genome assembly was conducted using SeqMan in Lasergene (version 7.1, DNASTAR Inc., Madison, WI, United States). The genome and the deduced amino acid sequences from the NSP2 or ORF5 gene alignments were assessed using ClustalW in Lasergene 7.1. The phylogenetic trees were constructed using MEGA (version 6.0) with the neighbor-joining method based on the ORF5 gene.

To ensure that these had sufficient temporal structure in alignment for reliable rate estimation, we first performed a regression of root-to-tip genetic distances on the ML tree against exact sampling dates using the TempEst. The rate of NADC30-like PRRSV (L1.8) and HP-PRRSV (L8.7) were estimated using the Bayesian Markov chain Monte Carlo (MCMC) method, MCMC inference was performed using the GTR + γ model, which was selected as the best nucleotide substitution model of the data set estimated using ModelFinder under Phylosuite (Dokland, 2010; Kalyaanamoorthy et al., 2017). All analyses were performed with a Bayesian skyline model using a relaxed uncorrelated lognormal (UCLN) molecular clock. The MCMC algorithm was run for 200 million steps and sampled every 20,000 steps. Convergence was assessed with effective sample size (ESS) values, and ESS values over 200 were considered adequate. The effective population size was evaluated after 10% of the chain was discarded as burn-in. These analyses were performed using BEAST (v1.10.4; Suchard et al., 2018). Three independent runs were performed in this study to prevent any local convergence. The BEAST results were entered into Tracer to evaluate model convergence and consistency between replicates. We constructed a maximum-likelihood (ML) phylogeny using IQ-TREE (Nguyen et al., 2015), making use of ultrafast bootstrap (UFBoot; Hoang et al., 2018) to compute 1,000 bootstrap replicates. ClusterPicker software was used to further divide the lineage into sub-lineages according to the previous study (Ragonnet-Cronin et al., 2013; Paploski et al., 2019; Yu et al., 2020).

### Recombination Analysis

To analyze the recombination events of PRRSVs from the investigated pig producer, RDP v4.16 and the NCBI BLAST results were used to identify the major and minor parental strains. Then, the data were analyzed with Simplot (version 3.5.1) by advancing a 500-bp sliding window along the genome alignments with a 20-bp step size (Zhao et al., 2015; Zhang et al., 2018). Finally, the parental sequence and recombination fragment were further validated using a phylogeny tree generated based on the neighbor-joining method.

## Results

### Four Coexisting PRRSV Lineages (1, 3, 5, and 8) Were Associated With the Investigated Pig Producer

**Figure 1:**
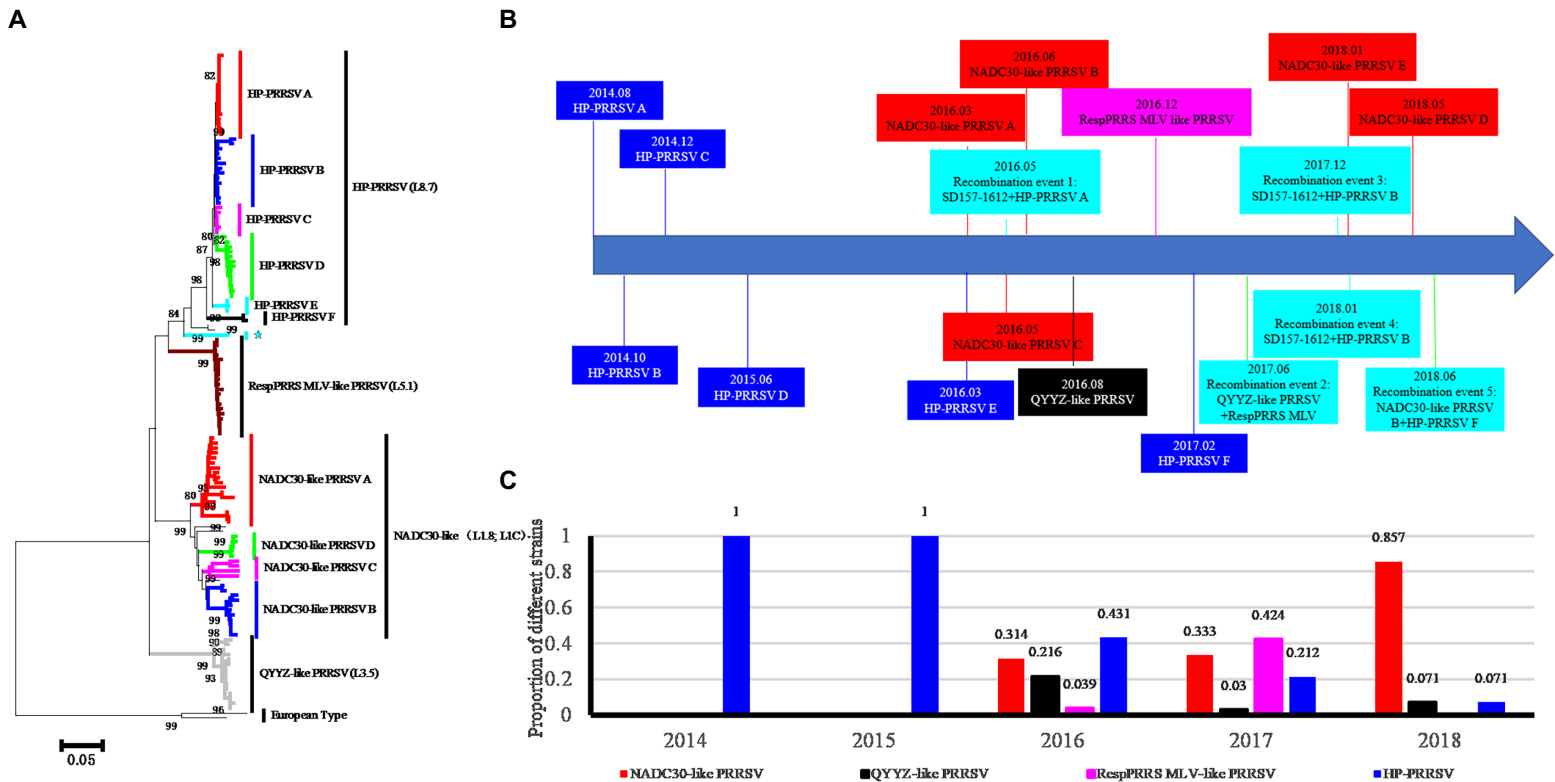
Phylogenetic clustering, emergence time and ratio of Porcine reproductive and respiratory syndrome virus (PRRSVs) sampled from a large-scale pig farm from 2014 to 2018. **(A)** Phylogenetic analysis of PRRSVs from the investigated pig producer. NADC30-like PRRSV E group recombined with HP-PRRSV in the ORF5 gene, causing this strain to become more similar to HP-PRRSV (indicated by ★). **(B)** Diagram of timeline for the emergence of novel PRRSVs. The emergence times of six HP-PRRSV groups and five NADC30-like PRRSV groups are shown in blue and red backgrounds, respectively. The occurrence times of QYYZ-like and RespPRRS MLV-like PRRSVs are shown with black and pink backgrounds, respectively. Five recombination events are indicated with a water green background. **(C)** The ratio of the four types of PRRSVs from the pig producer from 2014 to 2018.

To investigate the types of PRRSVs associated with the investigated pig producer, we sequenced 134 PRRSVs (including 121 ORF5 genes, 118 NSP2 genes, and 35 complete genomes; GenBank MH651744, MH651745, and MT093739-MT093771) from 338 suspected PRRSV-infected samples from August 2014 to October 2018. Phylogenetic and statistical analyses showed that four PRRSV lineages coexisted in the investigated pig producer (Figure 1A). Five groups in lineage 1 (NADC30-like) and six groups in lineage 8 (HP-PRRSV) were detected and classified based on phylogenetic tree and amino acids characteristics of ORF5 (Figure 1A; Supplementary Figure S1). Only lineage 8 PRRSV was detected before March 2016 (Figure 1B), while lineages 1, 3 (QYYZ-like), and 5 (RespPRRS MLV-like) were first detected in March, August, and December 2016, respectively (Figure 1B). In addition, we identified five novel recombination events among PRRSVs from the investigated pig producer (Figure 1B). The positive rate of lineage 8 and 1 PRRSVs decreased and increased over 4 years, respectively (Figure 1C), with lineage 1 PRRSVs replacing lineage 8 as the major epidemic strain in 2017 (Figure 1C).

### Importation, Variation, and Recombination Contribute to the Emergence of Novel PRRSVs

To elucidate the reason for the emergence of the novel lineage 8 PRRSVs, 53 ORF5 sequences of lineage 8 PRRSVs were sequenced over 4 years. Phylogenetic analysis and sequence alignment results showed that lineage 8 PRRSVs from the investigated pig producer were classified into six groups (HP-PRRSV A-F; Figure 1A; Supplementary Figure S1). We further sequenced six representative complete genome sequences of six HP-PRRSV groups. A sequence alignment showed that the NSP2 proteins from the six groups harbor a discontinuous 30-aa deletion consistent with HP-PRRSV in 2006 (Supplementary Figure S1). HP-PRRSV A (SD180-1702) and B (SD172-1702), which emerged before 2015, exhibited the highest similarity (whole genome) with FZ06A (99.32%) and GDHY (99.68%; Table 1), suggesting that HP-PRRSV A and B may have been imported into the investigated pig producer before August 2015. HP-PRRSV C-F have higher similarity (Table 1) with HP-PRRSV B and harbor the same amino acid mutation in the GP5 protein (Supplementary Figure S1), suggesting that these four HP-PRRSV groups may have evolved from HP-PRRSV B with different degrees of variation.

**Table 1:** Similarity comparison of whole genome among six HP-PRRSV groups.

PRRSV	SD180-1702	SD172-1702	SD23-1505	SD54-1603	SD65-1603	SD173-1702
SD180-1702 (HP-PRRSV A)	/	/	/	/	/	/
SD172-1702 (HP-PRRSV B)	98.7	/	/	/	/	/
SD23-1505 (HP-PRRSV C)	98.0	98.6	/	/	/	/
SD54-1603 (HP-PRRSV D)	97.3	97.9	97.2	/	/	/
SD65-1603 (HP-PRRSV E)	94.8	95.2	94.7	93.9	/	/
SD173-1702 (HP-PRRSV F)	95.1	95.1	94.7	93.9	92.2	/
Highest similarity strains in GenBank or in this study	FZ06A (99.32)	GDHY (99.68)	SD172-1702 (98.6)	SD172-1702 (97.9)	SD172-1702 (95.2)	SD172-1702 (95.1)

NADC30-like PRRSVs were identified in the investigated pig producer in March 2016. To elucidate the reason for the emergence of novel lineage 1 PRRSVs, 38 NADC30-like PRRSV strains were sequenced from March 2016 to August 2018. Phylogenetic analysis and sequence alignment results revealed the presence of five groups of NADC30-like PRRSVs (A–F) in the investigated pig producer (Figure 1A; Supplementary Figure S1). The sequence alignment results revealed that most of the NADC30-like PRRSVs harbored a discontinuous 131-aa deletion consistent with the NADC30 strain isolated in 2008 (Supplementary Figure S1), and almost all of the NADC30-like PRRSVs were recombination viruses. NADC30-like PRRSV A (SD53-1603), B (SD99-1606), D (SD288-1805), and E (SD265-1801) have the same recombination patterns and the highest similarity with QHD1 (98.18), SDZC1609 (95.77), 15JHEN1 (94.60), and SCN17 (99.62), respectively (Table 2), suggesting that NADC30-like PRRSV A, B, D, and E may have been imported into the investigated pig producer, after which a degree of variation occurred. Fortunately, during the course of the epidemiological investigation of the cooperative pig farms, we observed that NADC30-like PRRSV C (SD85-1605) was the result of recombination between SD157-1612 (detected from cooperative pig farms) and HP-PRRSV A.

**Table 2:** Similarity comparison of whole genome among six NADC30-like PRRSVs.

PRRSV	SD53-1603	SD99-1606	SD85-1605	SD288-1805	SD265-1801
SD53-1603 (NADC30-like PRRSV A)	/	/	/	/	/
SD99-1606 (NADC30-like PRRSV B)	94.2	/	/	/	/
SD85-1605 (NADC30-like PRRSV C)	84.2	84.7	/	/	/
SD288-1805 (NADC30-like PRRSV D)	90.1	90.3	85.8	/	/
SD265-1801 (NADC30-like PRRSV E)	90.7	91.7	85.5	88.8	/
Highest similarity strains in GenBank	QHD1 (98.18)	SDZC1609 (95.77)	15HEB1 (95.99)	15HEN1 (94.60)	SCN17 (99.62)
Recombination patterns	Y	Y	N	Y	Y

### Evolutionary Characteristic of Four Lineages PRRSV From the Investigated Pig Producer

To explore the evolutionary characteristics of PRRSVs from the investigated pig producer, the mutation and recombination characteristics of the four PRRSV lineages were analyzed. NADC30-like PRRSV has a faster mutation rate [7.55 × 10^−3^/site/year, 95% highest posterior density (2.90 × 10^−3^ ~ 1.26 × 10^−2^)] than that of HP-PRRSV [5.22 × 10^−3^/site/year, 95% highest posterior density (1.75 × 10^−3^ ~ 8.74 × 10^−3^)], which may be the primary causes of changes in predominant strains over short periods of time. We detected multiple lineage 1 PRRSVs that recombined with circulating strains (HP-PRRSV and RespPRRS MLV-like PRRSV) from the investigated pig producer. Lineage 3 PRRSVs have a higher recombination rate but lower mutation rate, and we observed that the recombinant virus SD110-1608 (one of the earliest lineage 3 PRRSVs) further recombined with the RespPRRS MLV-like PRRSV circulating in this farm. However, the nucleotide similarity of ORF5 for this virus over 2 years was 98–100%, indicating that lineage 3 PRRSVs have a lower mutation rate. In addition, all of the RespPRRS MLV-like PRRSVs with 98.8 ~ 100% similarity to the RespPRRS MLV strain belong to lineage 5, which had a low mutation rate. Last, lineage 8 PRRSVs emerged the earliest and evolved into multiple variant strains with a lower mutation rate [5.22 × 10^−3^/site/year, 95% highest posterior density (1.75 × 10^−3^ ~ 8.74 × 10^−3^)]. Interestingly, we did not identify recombination events before lineage 1 and 3 PRRSVs appeared. Therefore, lineage 1 and 3 PRRSVs are the root resulting in recombination, as most of them are the major parental strains of recombinant viruses. HP-PRRSV or RespPRRS MLV PRRSV provided the recombinant fragments (minor parental strains).

### Evolution of the Same NADC30-Like PRRSV Strain Over 2 Years

**Figure 2:**
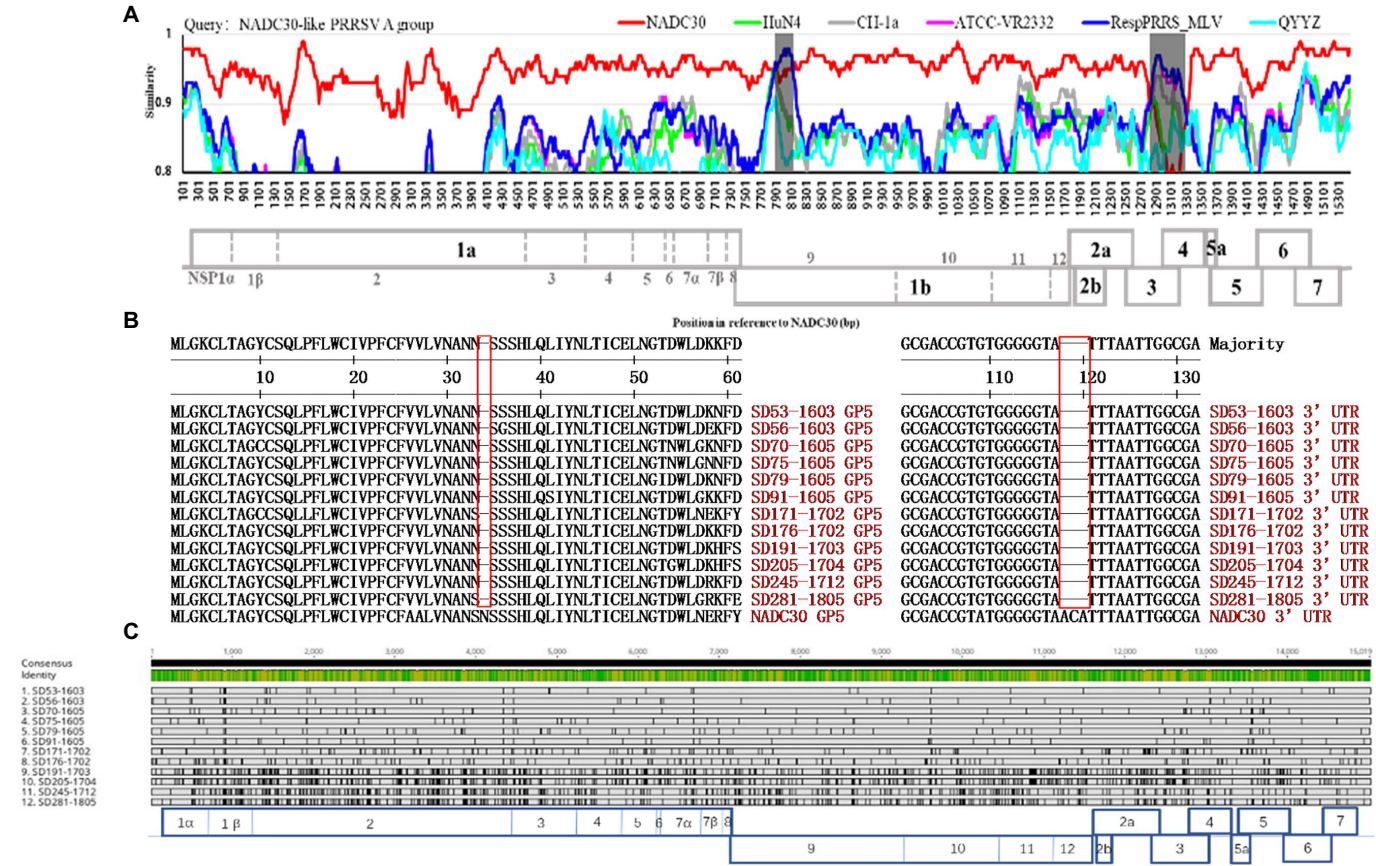
Recombination and evolutionary analysis of the same PRRSV strain sampled from a large-scale pig farm from 2014 to 2018. **(A)** The strains in the NADC30-like PRRSV A group shared the same recombination patterns. **(B)** Sequence alignment of GP5 and the 3′-UTR of strains in the NADC30-like PRRSV A group. **(C)** Schematic of the 12 NADC30-like PRRSVs in group A evolved from the same strain relative to the overall consensus from at least 50% of the sequences. Lightly shaded regions show similar identities to the consensus, and the vertical black bars show differences from the consensus (the alignment was completed using Geneious, https://www.geneious.com/).

The low genomic similarity among the NADC30-like PRRSVs made the evolutionary relationships of these viruses unclear. We sequenced 12 complete genomes from the NADC30-like PRRSV A group and analyzed their evolutionary characteristics. The NADC30-like PRRSV A group evolved from the same PRRS strain and shared the same recombination (Figure 2A) and deleted patterns (Figure 2B). Surprisingly, the same strain evolved at an astonishing rate over 2 years (Figure 2C). A sequence divergence of up to approximately 10% was observed for the some gene fragment, such as NSP7β, NSP8, and ORF3 (Supplementary Table S1). In addition, we observed that the ORF5 gene was the most susceptible in PSSRV to mutation (Supplementary Table S1). Furthermore, the degree of variation observed among all PSSRV proteins over 2 years followed the order of GP3 (90.6%) > GP5 > Nsp8 > Nsp7β > Nsp1β > Nsp2 > E > Nsp5 > GP2a > N > Nsp11 > GP4 > ORF5a > Nsp7α > Nsp3 > M > Nsp12 > Nsp4 > Nsp9 > Nsp10 > Nsp6 (100%; Supplementary Table S1). The UTRs were relatively conserved (3′UTR > 5′UTR; Supplementary Table S1). This is the first report describing the variation in the same PRRSV strain evolved in a pig herd. Our results revealed that the low genomic similarity of NADC30-like PRRSVs results from rapid mutation, which is one of the reasons that the evolutionary relationship of these viruses in China could not be elucidated.

### Complex PRRSV Recombination Patterns Formed Gradually

**Figure 3:**
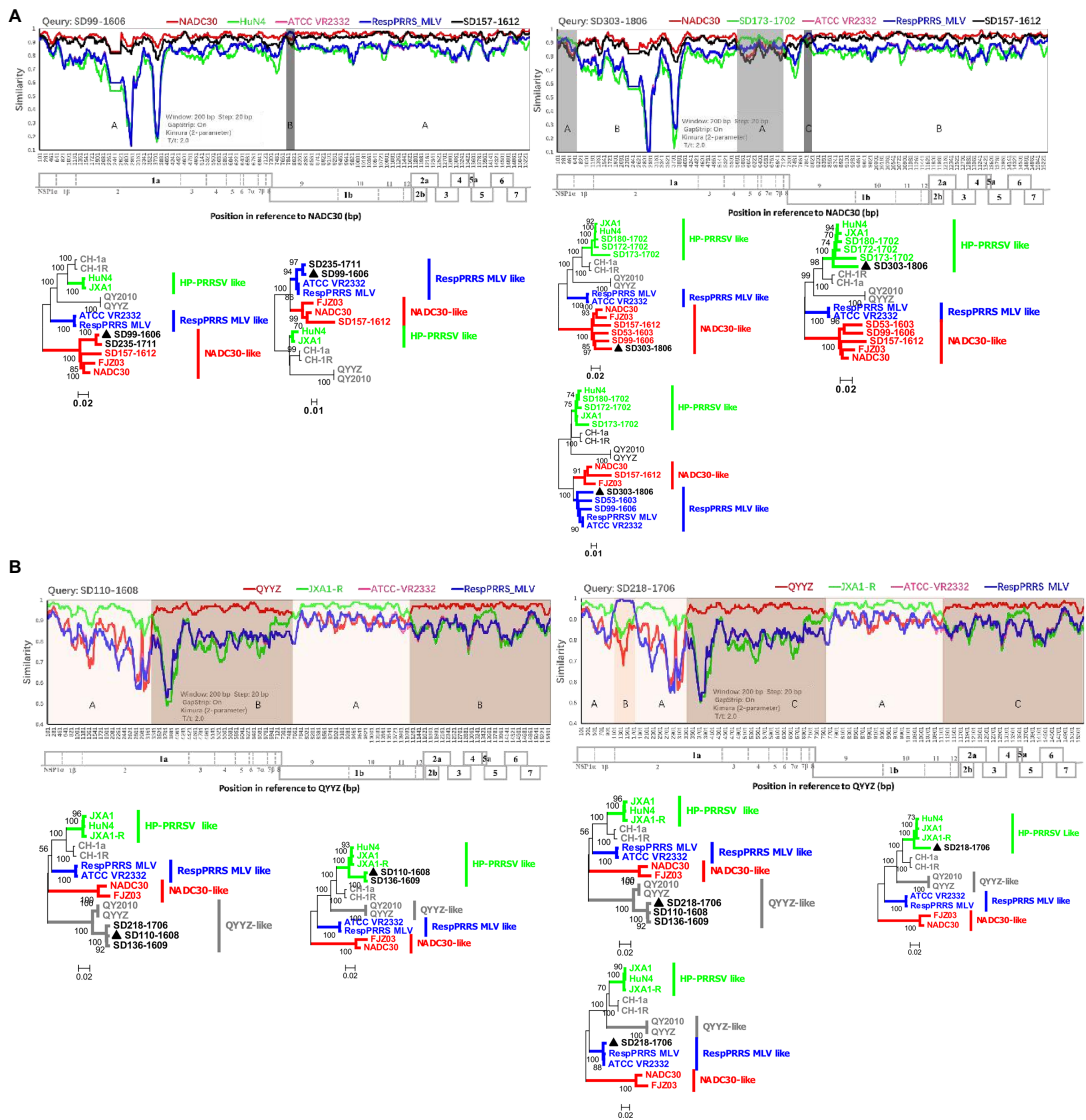
Complex recombination patterns formed gradually. **(A)** Recombination analysis of the NADC30-like PRRSV strains SD99-1606 and SD303-1806. Reference strains (NADC30, HuN4 or SD173-1702, ATCC-VR2332, RespPRRS MLV, and SD157-1612) are shown in red, green, pink, blue, and black, respectively. **(B)** Recombination analysis of strains SD110-1608 and SD218-1706 in lineage 3. Reference strains (QYYZ, JXA1-R, ATCC-VR2332, and RespPRRS MLV) are shown in red, green, pink, and blue, respectively. Recombination analysis was performed using Simplot 3.5.1. Below the similarity plots is shown the complete genome structure of PRRSV (with reference to strains NADC30 and QYYZ), where the positions of the 10 open reading frames and the 14 non-structural proteins are shown. Recombination breakpoints and their locations are shown at the bottom. The phylogenies of major and minor parental regions are shown below the similarity plots.

Few recombination events among PRRSVs occurred before 1991–2013 in China (Yu et al., 2020), but complex recombination patterns of these viruses have been reported in recent years. To explore how the complex recombination patterns currently observed formed, we identified and compared numerous recombinant viruses from the investigated pig producer. The recombination analysis results showed that 24 of 35 complete genomes (HP-PRRSV 0/6; NADC30-like PRRSV 20/21; QYYZ-like PRRSV 4/4; and RespPRRS MLV-like PRRSV 0/4) were recombinant viruses. No recombinant event was detected before the emergence of NADC30-like and QYYZ-like PRRSVs. However, since the emergence of both strains, all related strains identified from the investigated pig producer except one have been recombinant viruses. Furthermore, in June 2016, we identified a strain (SD99-1606, NADC30-like PRRSV B) resulting from recombination between NADC30 and RespPRRS MLV from this pig farm (Figure 3A). After 2 years, we detected another strain (SD303-1806, NADC30-like PRRSV B) resulting from recombination between SD99-1606 and SD173-1702 (HP-PRRSV F; Figure 3A). Similarly, in August 2016, we identified a strain (SD110-1608, QYYZ-like PRRSV) from the same pig farm that was a recombinant virus between QYYZ and JXA1 (Figure 3B). In June 2017, we identified another strain (SD218-1706, QYYZ-like PRRSV) from the same pig farm that was a recombinant virus between SD110-1608 and RespPRRS MLV-like PRRSV (Figure 3B). All of these results showed that NADC30-like and QYYZ-like PRRSVs are more prone to recombination and that the complex recombination patterns observed formed gradually. Furthermore, we speculate that these complex recombination patterns will increase for the foreseeable future until a predominant population forms and outbreaks.

### Recombination of the Same Strain Can Occur at Different Locations and Increase the Diversity of Recombination Events

**Figure 4:**
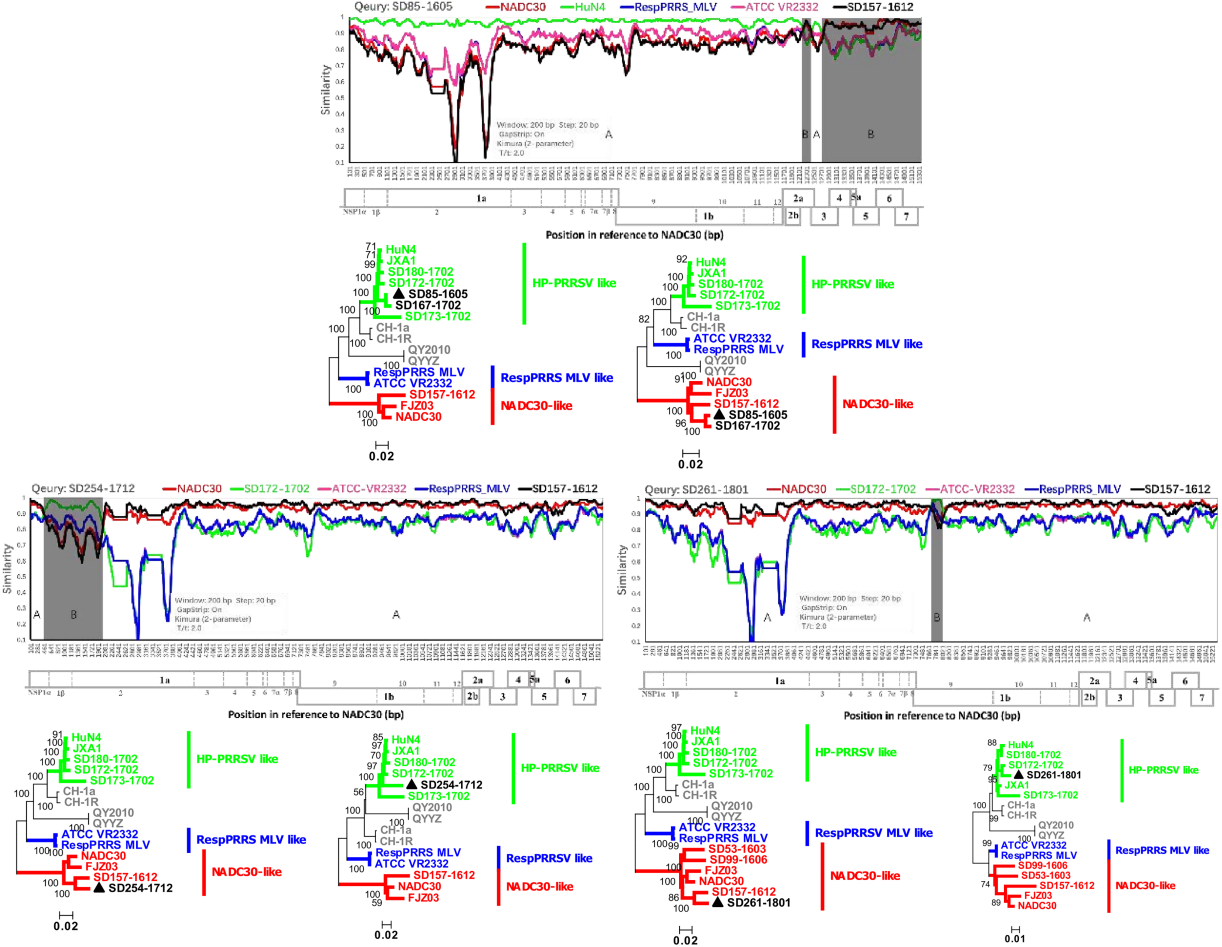
Recombination analysis of three NADC30-like PRRSVs with the same parental strain SD157-1612. Reference strains (NADC30, HuN4 or SD172-1702, ATCC-VR2332, RespPRRS MLV, and SD157-1612) are shown in red, green, pink, blue, and black, respectively. Recombination analysis was performed using Simplot 3.5.1. Below the similarity plots is the complete genome structure of PRRSV (with reference to strain NADC30), where the positions of the 10 open reading frames and the 14 non-structural proteins are shown. Recombination breakpoints and their locations are shown at the bottom. The phylogenies of major and minor parental regions are shown below the similarity plots.

The observed diversity of recombination events among PRRSVs since NADC30-like PRRSVs were detected in the investigated pig producer in March 2016 was perplexing. Fortunately, we detected one NADC30-like isolate (SD157-1612) with no recombination events from cooperative pig farms, which provided gilts for the investigated pig producer. Both recombination viruses (SD85-1605 and SD167-1702) are the result of recombination between SD157-1612 and HP-PRRSV A (Figure 4). In December 2017 and January 2018, we detected another two novel NADC30-like isolates (SD254-1712 and SD261-1801), which have SD157-1612 as the major parental strain and SD172-1702 (HP-PRRSV B) as a minor parental strain (Figure 4). Our data provide direct evidence that importation and further recombination at different locations among genomes with circulating strains occurred, increasing the diversity of recombinant strains.

## Discussion

In China, type 2 PRRSVs are the predominant strains, and at least five lineages (including lineages 1, 3, 5, 8, and 9) have circulated from 1996 to 2016 (Gao et al., 2017; Guo et al., 2018). In the present study, we detected four PRRSV lineages (1, 3, 5, and 8) from the investigated pig producer, allowing for the evolution of PRRSVs to be monitored in a relatively closed environment. The results of a previous study showed that the prevailing PRRSV rapidly changed from type 2 to type 1 in 2 weeks after the introduction of type 1 PRRSVs over a 1-year study in a swine farm in Korea (Kim et al., 2011). However, we cannot explain how these novel PRRSVs emerged and what their relationships are in China. In the present study, we report the origin and evolution of a novel PRRSV strain in a large-scale pig producer and the evolution of the same strain in the swine herd through long-term genome monitoring over 4 years.

NADC30-like PRRSVs have been reported to originate from the United States (Sun et al., 2020; Yu et al., 2020). However, the origin of recently identified novel strains (e.g., NADC30-like and NADC34-like) has remained unclear because of low genomic similarity and complex recombination patterns. In the present study, we identified the source of early PRRSVs among different groups from the investigated pig producer. The phylogenetic analysis, emergence time, GP5 amino acid alignment, and complete genome nucleotide similarity results suggested that HP-PRRSV A-B existed before 2014 and may have been imported from outside of the investigated pig producer. In addition, the highly complex and diverse recombinant patterns of NADC30-like PRRSV made it extremely difficult to identify PRRSV isolates from different pig farms with the same recombination patterns. NADC30-like PRRSVs were identified in the investigated pig producer in March 2016. NADC30-like PRRSV A, B, D, and E have the same recombination patterns and the highest similarity with QHD1, SDZC1609, 15JHEN1, and SCN17, respectively, suggesting that NADC30-like PRRSV A, B, D, and E may have been imported into the investigated pig producer at different times. Finally, in August 2016, we detected a novel QYYZ-like PRRSV in the investigated pig producer. Although, we have not identified a PRRSV in GenBank with high nucleotide similarity and the same recombination patterns as the QYYZ-like PRRSV identified in the present study pig producer, we considered that these QYYZ-like PRRSVs may also have been imported from other pig farms based on the continuous monitoring for PRRSV by the investigated pig producer. In addition, our lab and another research group have confirmed that QYYZ-like PRRSVs of mainland China originated from Taiwan (Sun et al., 2018; Zhang et al., 2019), and the QYYZ-like PRRSVs from the investigated pig producer were likely from Guangdong Province (Zhang et al., 2019). Over the course of studying the origin of PRRSV in the investigated pig producer, we also detected PRRSV in gilt farms of cooperative pig farms. Interestingly, we identified one NADC30-like PRRS strain (SD157-1612) with no recombination in above gilt farm in December 2016, indicating that SD157-1612 and HP-PRRSV A or B in this pig farm recombined to form a new NADC30-like PRRSV C strain. Although how these novel PRRSVs were imported into the investigated pig producer remains unclear, these results show that importation is indeed an important route for the emergence of novel PRRSVs.

Mutations cause variation in PRRSVs and lead to the evolution of novel viruses or new groups. The results of a previous study demonstrated that HP-PRRSV evolved from classical PRRSV in China by deletion and mutation (An et al., 2010). In the present study, HP-PRRSV C-F have high similarity and the same amino acid mutations as HP-PRRSV B, suggesting that these four HP-PRRSV groups most likely evolved from HP-PRRSV B. In addition, the NADC30-like PRRSVs emerged and circulated in China since 2013. However, the low levels of whole-genome similarity and a wide variety of recombination patterns obscured their transmission between different pig farms. Luckily, different strains among NADC30-like PRRSVs from the investigated pig producer exhibited obvious characteristics, such as recombination and deletion in specific proteins or genes. Furthermore, we observed that NADC30-like PRRSV A group strains, which evolved from the same strain, had become the dominant strains and that the amino acid similarity of their GP3 proteins decreased to 90.6%. Thus, these novel and highly mutated NADC30-like PRRS strains are closely related to an early NADC30-like PRRSV.

There are few reports describing PRRSV recombination before the emergence of NADC30-like and QYYZ-like PRRSVs. Recombination analysis results showed that 24 of 35 complete genomes were recombination viruses. Before March 2016, all the PRRSVs detected in the investigated pig producer lacked recombination events. However, since the emergence of NADC30-like and QYYZ-like PRRSVs, all related strains in the investigated pig producer were recombination viruses. Furthermore, the primary backbone of the recombination viruses was NADC30-like or QYYZ-like PRRSVs. We consider that recombination events primarily caused these two type strains. In addition, strains SD85-1605, SD167-1702, SD254-1712, and SD261-1801 resulted from recombination between SD157-1612 and HP-PRRSV (A or B), suggesting that the same strain recombined with the circulating strains in the investigated pig producer at different locations, increasing PRRSV diversity. Although the recombination breakpoints occurred in NSPs (nsp9) and/or minor structural proteins (GP2a-GP3; Zhang et al., 2019; Yu et al., 2020), the recombination events exhibited a certain degree of randomness based on our monitoring results. Taken together, the above results showed that recombination is another means by which the diversity of PRRSV increases and promotes the emergence of new viruses.

Our group and other research groups have observed many novel PRRSVs formed by recombination with three or four PRRSVs lineages or by recombination at multiple locations in China in recent years (Zhou et al., 2018b; Liu et al., 2019). Low levels of whole-genome similarity and a wide variety of recombination patterns (Liu et al., 2019) complicate evolutionary analyses of PRRSV. Over the course of monitoring the same PRRSV strain, we identified two recombination events: the NADC30-like PRRSV strain SD303-1806 is the result of recombination between SD99-1606 and RespPRRS MLV-like PRRSV; and the QYYZ-like PRRSV strain SD218-1706 is the result of recombination between SD110-1608 and RespPRRS MLV-like PRRSV. The above results are direct evidence for PRRSV recombination. We identified two recombination viruses that further recombined with the epidemic strains from the investigated pig producer over 2 years, resulting in the complex PRRSV recombination patterns currently observed. Furthermore, our results demonstrated that these complex recombination patterns of PRRSVs formed gradually.

## Conclusion

In summary, various types of PRRSVs coexisted in the same pig farm investigated in the present study, increasing the probability of their recombination. Importation, variation, and recombination have caused the emergence of novel PRRSVs. NADC30-like PRRSVs, which have a high mutation rate, have become major epidemic strains in a short period of time. The current complex recombination patterns of PRRSV formed gradually. Recombination of the same strain occurs at different locations and increase the diversity of recombination events. Our findings provide direct evidence for the emergence and evolution of PRRSVs and will promote a better understanding of evolution of viruses.

## Data Availability Statement

The original contributions presented in the study are included in the article/Supplementary Material, further inquiries can be directed to the corresponding author.

## Author Contributions

Z-JT and HZ: conceptualization. LX, Z-JT, and HZ: data curation. Z-JT, HZ, and T-QA: funding acquisition. LX, HX, CLi, Y-DT, and T-QA: methodology. ZL, CLiu, SS, JZ, CLe, XQ, YS, JP, and QW: resources. LX, CLi, and SS: software. Y-DT, T-QA, and XC: supervision. HX: validation. LX and HX: visualization. LX: writing—original draft and writing—review and editing. All authors contributed to the article and approved the submitted version.

## Funding

The study was supported by grants from the National Natural Science Foundation of China (Grant nos. 32002315 and 32172890), the China Postdoctoral Fund (Grant no. 2020M680788), the Harbin Science and Technology Foundation for Innovative Talents (2016RAQXJ142), the Heilongjiang Natural Science Foundation for Distinguished Young Scholars (JC2017010), and the State Key Laboratory of Veterinary Biotechnology Science Fund (SKLVBF201902).

## Conflict of Interest

XQ and YS are employed by Hanswine FoodGroupCo.

The remaining authors declare that the research was conducted in the absence of any commercial or financial relationships that could be construed as a potential conflict of interest.

## Publisher’s Note

All claims expressed in this article are solely those of the authors and do not necessarily represent those of their affiliated organizations, or those of the publisher, the editors and the reviewers. Any product that may be evaluated in this article, or claim that may be made by its manufacturer, is not guaranteed or endorsed by the publisher.
