# Supplementary material for: Long-Term Genome Monitoring Retraces the Evolution of Novel Emerging Porcine Reproductive and Respiratory Syndrome Viruses
Source: Front Microbiol. 2022 Apr 13;13:885015. doi: 10.3389/fmicb.2022.885015 (PMC9044490; doi:10.3389/fmicb.2022.885015)
Supplement: Supplementary file 1 [file Table_1.DOCX]

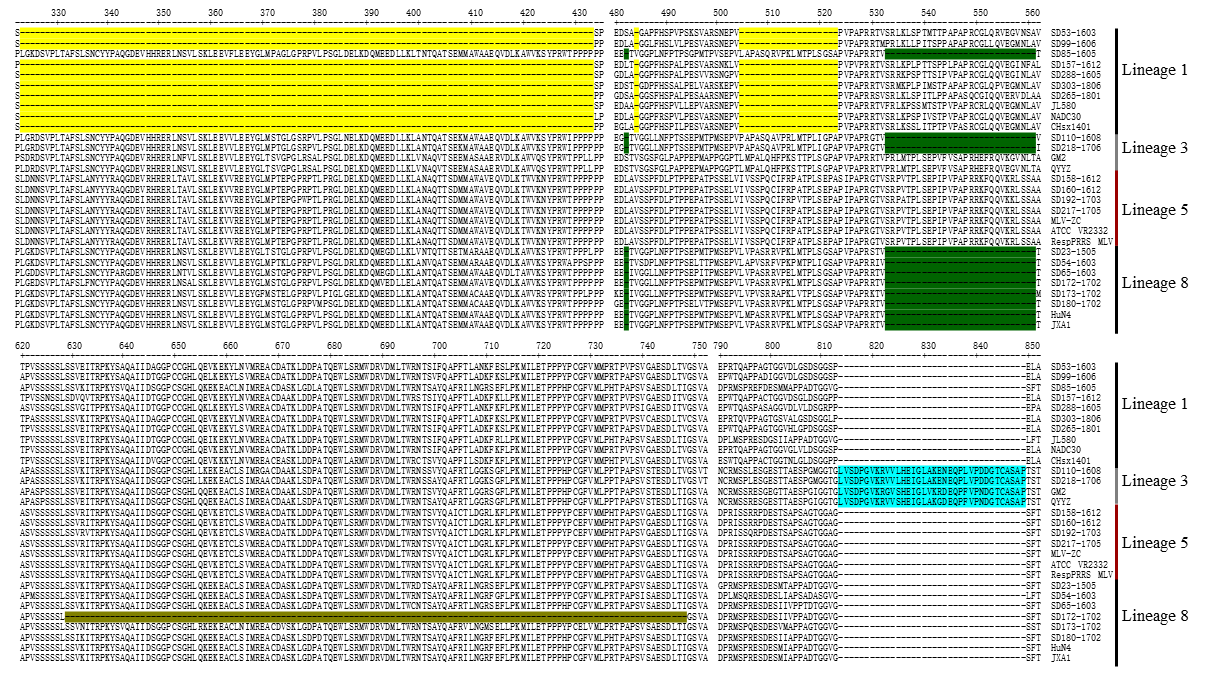


(B)

(A)


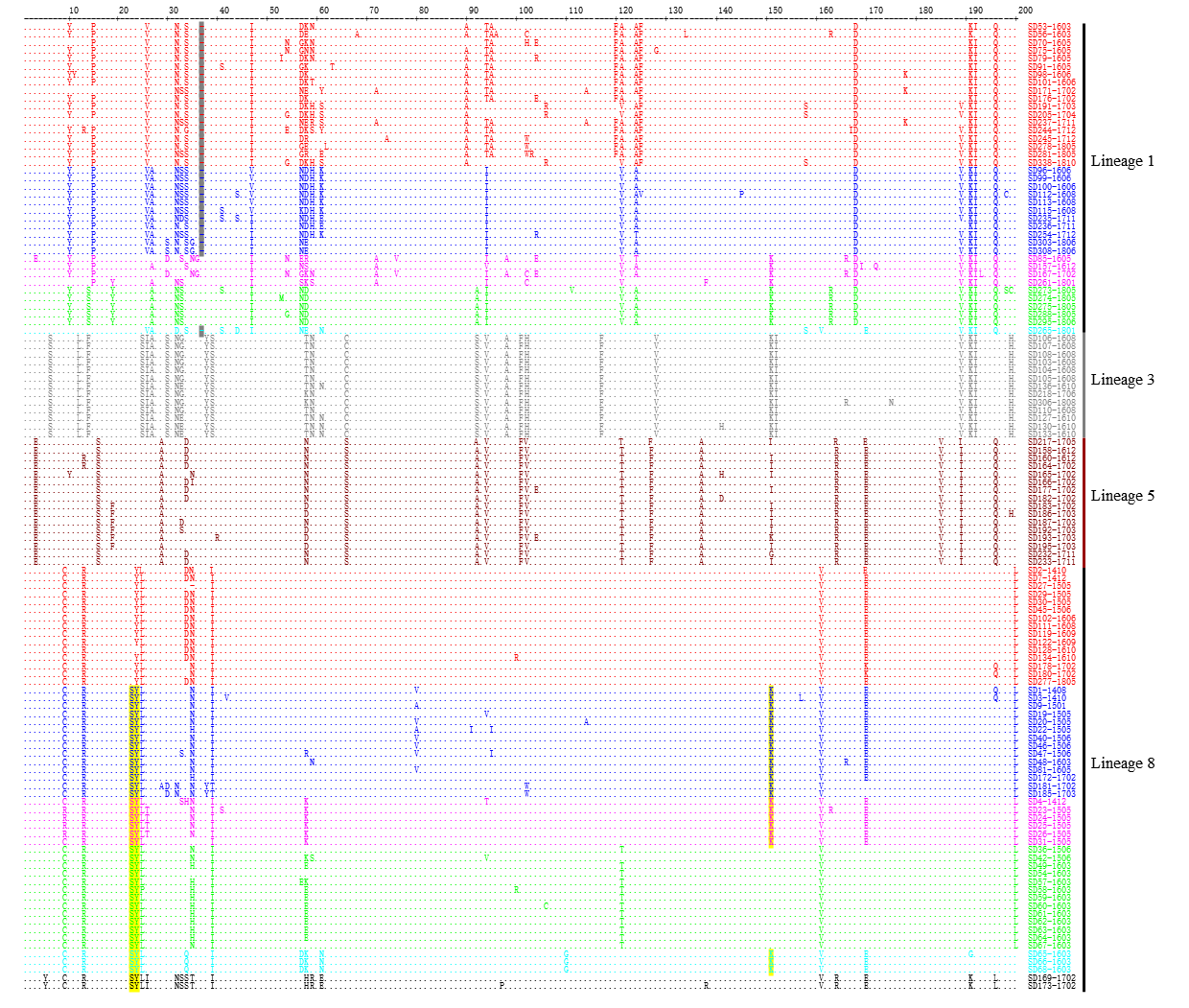


**FIGURE S1. Deduced NSP2 and GP5 amino acid sequence alignment for lineage 1 (NADC30-like PRRSV), lineage 3 (QYYZ-like PRRSV), lineage 5 (RespPRRS MLV-like PRRSV) and lineage 8 (HP-PRRSV).** **(A)** Deduced amino acid sequence alignment of NSP2 protein. The NSP2 protein of lineage 1 (NADC30-like PRRSVs) has a discontinuous 131-amino-acid deletion indicated with yellow background; the NSP2 protein of lineage 3 (QYYZ-like PRRSVs) has a discontinuous 30-amino-acid deletion and a continuous 36-amino acid insertion indicated with green and water green backgrounds, respectively; the NSP2 protein of lineage 8 (HP-PRRSVs) has a discontinuous 30-amino-acid deletion, which is indicated with a green background, SD172-1702 has an additional 120 consecutive amino acid deletions, which is indicated with a dark yellow background. **(B)** Deduced amino acid sequence alignment of GP5 protein. Different groups of lineage 1, 3, 5 and 8 represented by different colors. Key amino acid mutations or deletions were labeled with different background colors.
