# Supplementary material for: Long-Term Genome Monitoring Retraces the Evolution of Novel Emerging Porcine Reproductive and Respiratory Syndrome Viruses
Source: Front Microbiol. 2022 Apr 13;13:885015. doi: 10.3389/fmicb.2022.885015 (PMC9044490; doi:10.3389/fmicb.2022.885015)
Supplement: Supplementary file 2 [file Table_2.DOCX]

Table S1. Sequence alignment of NADC30-like PRRSV A groups

| Gene | The emerging time of NADC30-like PRRSV A strains (12 strains) | | | | | | |
| --- | --- | --- | --- | --- | --- | --- | --- |
|  | March 2016 | May 2016 | February 2017 | March 2017 | April 2017 | December 2017 | May 2018 |
| 5’UTR | 98.9 | 98.4-98.9 | 97.4-99.5 | 97.4-99.5 | 97.4-100 | 98.4-100 | 97.4-98.9 |
| Nsp1α | 100/100 | 98.7-99.4/98.9-99.4 | 98.0-99.4/98.3-100 | 95.7-96.5/95.6-97.2 | 95.7-100/95.6-100 | 94.8-98.1/95.6-98.3 | 94.8-100/95.6-100 |
| Nsp1β | 99.8/99.5 | 98.8-99.5/97.5-98.5 | 97.5-99.8/96.5-99.5 | 95.4-97.0/96.0-98.0 | 95.2-99.8/95.5-99.5 | 94.2-95.5/**92.6**-95.0 | 94.4-99.8/**92.6**-100 |
| Nsp2 | 99.7/99.6 | 99.1-99.5/99.0-99.6 | 98.5-98.9/98.0-98.6 | 95.3-96.0/**93.9-94.8** | 95.2-99.5/**93.5**-99.2 | 94.7-95.7/**93.2**-94.7 | 94.6-99.7/**93.1**-99.3 |
| Nsp3 | 99.7/100 | 99.1-100/99.1-100 | 98.1-99.6/98.3-99.6 | 96.2-97.2/97.0-98.3 | 96.2-99.1/97.4-99.6 | 96.1-97.8/96.5-99.1 | 96.1-99.7/96.5-100 |
| Nsp4 | 99.5/99.0 | 99.2-99.8/99.0-100 | 98.9-99.2/98.5-100 | 96.7-97.4/98.0-99.0 | 96.6-99.8/98.0-100 | 97.1-97.5/98.0-99.0 | 97.1-100/98.0-100 |
| Nsp5 | 99.2/99.4 | 98.8-99.6/98.8-100 | 98.0-99.0/98.2-99.4 | 95.9-96.7/95.9-96.5 | 95.9-100/95.9-100 | 95.1-98.0/**94.1**-97.6 | 95.1-100/**94.1**-100 |
| Nsp6 | 100/100 | 100/100 | 97.9-100/100 | 91.7-93.8/100 | 91.7-100/100 | 93.8-100/100 | 93.8-100/100 |
| Nsp7α | 99.8/100 | 99.3-100/100 | 98.2-98.9/98.0-99.3 | 96.6-97.5/96.0-96.6 | 96.9-99.3/96.0-98.7 | 96.9-98.0/96.6-98.7 | 96.6-99.8/96.6-100 |
| Nsp7β | 100/100 | 99.4-100/100 | 97.3-99.1/97.3-98.2 | 96.4-98.5/**92.7**-95.5 | 95.8-99.4/**91.8**-99.1 | 95.2-97.6/**91.8**-95.5 | 95.2-100/**91.8**-100 |
| Nsp8 | 99.3/97.8 | 98.5-100/95.6-100 | 97.8-100/95.6-100 | 94.8-96.3/**93.3**-95.6 | 94.8-100/**93.3**-100 | 93.3-96.3/**91.1**-95.6 | 92.6-99.3/**91.1**-97.8 |
| Nsp9 | 99.7/100 | 99.4-99.8/99.7-100 | 97.8-99.2/99.2-99.5 | 97.5-98/99.2-99.7 | 97.4-99.7/99.2-100 | 96.6-97.4/98.3-98.8 | 96.5-99.6/98.1-98.8 |
| Nsp10 | 99.9/100 | 99.5-99.9/99.8-100 | 98.6-99.5/99.5-100 | 96.7-97.3/99.5-99.8 | 96.7-99.9/99.5-100 | 96.4-97.5/99.3-99.5 | 96.1-99.6/98.9-99.5 |
| Nsp11 | 99.6/100 | 99.1-99.7/99.6-100 | 99.1-99.7/99.1-100 | 95.5-96.0/96.4-96.9 | 95.5-100/96.4-100 | 94.5-97.96/95.5-98.7 | 94.0-99.3/**94.6**-98.7 |
| Nsp12 | 99.8/99.3 | 99.6-100/99.3-100 | 99.1-99.8/97.4-100 | 96.7-97.2/98.0-98.7 | 96.9-99.8/98.0-100 | 96.3-98.5/98.7-99.3 | 96.3-100/98.7-100 |
| ORF2a | 99.9/99.6 | 99.6-100/99.2-100 | 96.2-99.6/95.7-99.6 | 94.0-96.5/**94.6**-96.5 | 94.0-100/**94.6**-100 | 95.5-98.2/95.3-96.9 | 94.7-99.1/**94.2**-98.8 |
| ORF2b | 100/100 | 99.5-100/98.6-100 | 97.3-99.1/95.9-98.6 | 96.4-97.7/**93.2**-95.9 | 96.4-100/**93.2**-100 | 96.8-98.2/**94.6**-98.6 | 96.4-99.5/**93.2**-98.6 |
| ORF3 | 99.6/99.6 | 98.8-99.5/ | 97.0-99.0/98.0-99.6 | 94.1-95.7/**94.1**-98.0 | 93.6-99.5/**91.0**-93.7 | 94.2-97.9/**90.6**-99.2 | 93.6-98.8/**91.0**-97.3 |
| ORF4 | 99.4/99.4 | 99.1-99.6/97.8-99.4 | 98.3-99.4/97.2-99.4 | 96.8-98.0/95.5-96.1 | 96.8-100/95.5-100 | 97.0-98.5/95.5-97.8 | 96.8-99.8/**95.0**-99.4 |
| ORF5 | 99.2/97.5 | 98.2-99.2/**95.0**-99.0 | 97.2-98.5/**93.5**-98.5 | 94.7-96.7/**91.5**-95.5 | 94.5-99.8/**91.0**-99.5 | 95.7-98.8/**94.5**-98.0 | 95.0-99.3/**93.0**-98.0 |
| ORF5a | 99.3/97.8 | 98.6-100/97.8-100 | 96.4-100/95.7-100 | 97.1-100/97.8-100 | 97.1-100/97.8-100 | 97.1-100/97.8-100 | 97.8-99.3/97.8-100 |
| ORF6 | 99.8/99.4 | 99.6-100/98.9-100 | 99.2-99.6/97.7-100 | 97.5-98.1/97.7-98.9 | 97.5-99.6/97.7-98.9 | 98.3-99.4/98.9-100 | 97.7-99.0/97.1-98.3 |
| ORF7 | 99.5/100 | 99.5-100/100 | 98.1-98.9/99.2-100 | 96.8-98.1/96.0-96.8 | 96.5-99.7/96.0-100 | 96.8-98.9/95.2-98.4 | 96.5-99.7/**94.4**-99.2 |
| 3’UTR | 100 | 99.3-100 | 98.6-100 | 99.3-100 | 98.6-99.3 | 98.6-99.3 | 98.6-100 |

Note: The nucleotide or deduced amino acid similarity were calculated using the current strains and their previous strains. For example, the strains in February 2017 compared with that of March and May 2016. Amino acid similarity ≤95.0% was defined as highly variable labeled with red colors.
